# Re‐evaluation of the dinosaur tail sign: Analysis of spinal magnetic resonance imaging signs in patients with chronic post–dural‐puncture headache

**DOI:** 10.1111/head.70210

**Published:** 2026-09-28

**Authors:** Charlotte Zander, Horst Urbach, Katharina Wolf, Amir El Rahal, Florian Volz, Christian Fung, Laura Krismer, Hansjörg Mast, Jürgen Beck, Niklas Lützen

**Affiliations:** ^1^ Department of Diagnostic and Interventional Radiology, Medical Center Faculty of Medicine, University of Freiburg Freiburg Germany; ^2^ Department of Neuroradiology, Medical Center Faculty of Medicine, University of Freiburg Freiburg Germany; ^3^ Department of Neurosurgery, Medical Center Faculty of Medicine, University of Freiburg Freiburg Germany; ^4^ Lindenhofspital Bern, Inselspital Bern Bern Switzerland

**Keywords:** arachnoid bleb, chronic post–dural‐puncture headache, CSF loss disorder, dinosaur tail sign, MR imaging, post‐dural‐puncture headache

## Abstract

**Objective:**

This study aimed to assess magnetic resonance imaging (MRI) findings of the lumbar spine in patients with chronic post–dural‐puncture headache.

**Background:**

Post–dural‐puncture headache is a common complication after dural puncture usually resolving within 14 days. However, a chronic course (>14 days persistence) is increasingly being recognized and may lead to tremendous symptom burden. While current research shows that MRI of the head is mostly unremarkable in chronic post–dural‐puncture headache, lumbar imaging signs have not yet been systematically studied.

**Methods:**

This is a retrospective, single‐center diagnostic case–control study. We identified all patients between 09/2022 and 12/2024 at the University Hospital Freiburg, Germany, with chronic post–dural‐puncture headache (defined as persisting symptoms for >14 days post puncture or after ≥1 epidural blood patch), and recruited 30 healthy controls between 10/2024 and 07/2025 without prior history of intentional or unintentional lumbar puncture, all undergoing 3D high‐resolution heavily T2‐weighted lumbar MRI (at 0.63 mm spatial resolution). The “dinosaur tail sign” (interspinous arched T2w‐hyperintensities) was assessed as originally published, recording numbers of involved spinal levels, while its appearance was subjectively graded as subtle, moderate, or prominent. Moreover, presence of arachnoid blebs and lumbar extradural fluid were recorded. Evaluation was independent and blinded; discrepancies were resolved by consensus. In a subgroup of patients with T1‐weighted contrast‐enhanced lumbar spine imaging (51/54), suspicious enhancement, which potentially may be attributable to the puncture, was analyzed, while none of the controls received contrast enhanced spinal MRI.

**Results:**

The final cohort resulted in 54 patients with chronic post–dural‐puncture headache with 44 females (81%) and a mean age of 38.2 years (SD ±9.8). The dinosaur tail sign was present in 27/54 patients (50%) and 21/30 controls (70%), with a numerically higher frequency in controls (70% vs. 50%, odds ratio [OR] 2.31, 95% confidence interval [CI] 0.83–6.84, *p* = 0.107, *p*‐adjusted = 0.240). The sign appeared at a median of two levels in both groups (interquartile range [IQR] 2.0–4.0 for patients vs. 1.0–3.0 for controls). Visual impression of the sign was graded subtle in 15 patients vs. 15 controls, moderate in nine vs. six, and prominent in three (two of three with extradural fluid) vs. none (*p* = 0.321, *p*‐adjusted = 0.432 for all groups). Lumbar extradural fluid was observed in 4/54 patients (7%), arachnoid blebs in 10/54 patients (19%; 9% definite, 9% suspicious), with one patient presenting with two suspected blebs. Neither extradural fluid nor arachnoid blebs were found in any controls. Overall, noticeable contrast enhancement in the lumbar spine was found in 17/51 patients (33%).

**Conclusion:**

The dinosaur tail sign, originally described in patients with acute post–dural‐puncture headache (≤14 days persistence), was unable to reliably distinguish patients with chronic post–dural‐puncture headache from healthy controls. Other findings, such as arachnoid blebs or extradural fluid collections, were only present in chronic post–dural‐puncture headache, but rare. Contrast enhancement in the lumbar spine was found in approximately one third of patients but was mostly attributed to nonspecific findings. Further spinal MRI signs need to be identified to enable diagnosis of chronic post–dural‐puncture headache in the future.

## Plain Language Summary

Certain features seen on magnetic resonance imaging (MRI) scans, such as the “dinosaur tail sign,” may play an important role in diagnosing patients with chronic complaints after lumbar puncture (i.e., chronic post–dural‐puncture headache) but this has not been systematically studied. We looked at MRI scans of patients with chronic post–dural‐puncture headache and compared them to healthy patients. While imaging features such as arachnoid blebs and lumbar fluid collections were rare but only seen in patients, the dinosaur tail sign was found in both healthy controls and symptomatic patients, highlighting that further research on this topic is needed.

**Abbreviations**
aOR: adjusted odds ratios
BMI: body mass index
CI: confidence interval
cPDPH: chronic post–dural‐puncture headache
CSF: cerebrospinal‐fluid
CT: computed tomography
CTM: CT myelography
EBP: epidural blood patch
FDR: false discovery rate
fs: fat saturated
GRAPPA: generalized autocalibrating partially parallel acquisitions
ICHD‐3: International Classification of Headache Disorders, 3rd edition
IQR: interquartile range
LP: lumbar puncture
MRI: magnetic resonance imaging
NPV: negative predictive value
OR: odds ratio
PDPH: post–dural‐puncture headache
PPV: positive predictive value
SD: standard deviation
SIH: spontaneous intracranial hypotension
SPACE: Sampling Perfection with Application‐optimized Contrasts using different flip angle Evolution
T1w: T1‐weighted
T2w: T2‐weighted
TSE: turbo spin echo
VIF: variance inflation factors

## INTRODUCTION

Post–dural‐puncture headache (PDPH) is a well‐known complication after lumbar puncture (LP) or unintended dural puncture (e.g., in terms of epidural anesthesia),^1^ occurring in up to 30%.^2^ According to the International Classification of Headache Disorders, 3rd edition (ICHD‐3), PDPH is described as a self‐limiting acute disease, resolving within 14 days.^3^ However, recent studies confirm the existence of a chronic course of PDPH (cPDPH) that can last for months or even years and may lead to tremendous symptom burden.^4^, ^5^, ^6^, ^7^, ^8^ This condition is often not properly recognized. In a recent study, a median of five consultations were needed before diagnosis was made resulting in approximately 1 month of acute inpatient care.^9^ Delaying the diagnosis leads to a delay in treatment, which in turn increases the risk of chronification.^9^, ^10^

Magnetic resonance imaging (MRI) could be helpful in identifying these patients and stratify further management; however, established cranial MRI features (the so‐called “Bern score”) used in patients with spontaneous intracranial hypotension (SIH) have shown to be largely unremarkable in patients with cPDPH.^8^, ^11^, ^12^ It is widely known that cranial MRI signs in patients with SIH can normalize over the course of the disease, which can lead to misdiagnosis, while imaging of the spine may still display epidural fluid, indicating a dural tear.^13^

Although previous studies demonstrated that only a small proportion of patients with cPDPH show fluid collections,^8^, ^14^ other spinal MRI imaging signs remain to be investigated: In 2017, the “dinosaur tail sign,” characterized by arched T2‐weighted (T2w) hyperintensities in the lumbar dorsal epidural fat tissue was described as a promising MRI marker of spinal CSF loss, but data on the occurrence and significance in cPDPH are lacking.^15^, ^16^ Moreover, small case series have described an “arachnoid bleb,” which represents a small herniation of arachnoid through the dural defect at the lumbar puncture site;^14^, ^17^, ^18^, ^19^ however, systematic analyses in cPDPH have not yet been performed. Finally, dural or nerval characteristic contrast enhancements, potentially indicating dural scar tissue at the lumbar puncture site, have not yet been investigated at all.^20^ Further insights into such alterations could thus contribute not only to an improved detection but may also help better understanding of underlying disease mechanisms.

In this study, we aimed to perform a systematic analysis of spinal MRI signs in a large cohort of patients with cPDPH compared to a group of healthy controls. Particular focus was put on the significance of the “dinosaur tail sign,” supplemented by evaluation of lumbar epidural fluid collections and “arachnoid blebs,” in order to identify patients with cPDPH on spinal MR imaging. We hypothesized that those spinal MRI signs would be more prevalent in patients with cPDPH compared to healthy controls. Furthermore, lumbar puncture could lead to local scarring, which may be visualizable analyzing local contrast enhancement.

## METHODS

### Study design

This is a retrospective, single‐center, two‐gate diagnostic case–control study based on a chart review with a post hoc primary analysis of all collected data. The study was approved by the local Institutional Review Board from the University Hospital Freiburg, Germany (24‐1296‐S1‐retro). Owing to the retrospective design, individual patient consent was waived.

The study furthermore consisted of a cohort of normal controls, all undergoing the same 3D high‐resolution heavily T2w lumbar MRI sequence as patients (24‐1396‐S1). All controls provided informed written consent.

### Study population

All patients with PDPH according to the ICHD‐3 criteria^3^ undergoing our standard imaging protocol for post–dural‐puncture headache using a 3‐Tesla MRI scanner were retrospectively identified. The protocol was established in our institution in September 2022. All patients investigated from then on until December 2024 were screened for inclusion criteria: (a) >18 years of age, (b) no prior operation in the lumbar spine, and (c) a chronic course of PDPH, which was defined as a history of intended or unintended dural puncture with subsequent symptoms suggestive of CSF loss, either persisting >14 days post puncture or persisting after one or more epidural blood patches (EBPs).

The control group was prospectively recruited between October 2024 and July 2025 from clinic staff and students. Inclusion criteria were age >18 years and the ability to provide oral and written informed consent. Exclusion criteria included any contraindication to MRI, any manipulation or intervention involving the spine and causing potential dural injury, and a history of or suspected CSF leak disorder. Recruitment from clinic staff resulted in a relatively young age distribution (<65 years), which is generally in line with the typically younger age of patients with PDPH. Sex was not used as a primary selection criterion; however, an effort was made to maintain a balanced sex distribution in order to account for potential sex‐specific differences. Participation was voluntary and uncompensated.

### Variables studied

Demographic data were extracted from the patients' charts and included: sex, age, and body mass index (BMI). The date of the dural puncture as well as data on prior EBP were taken from the patients' medical history. Where available, the exact date of symptom onset was used to calculate symptom duration. In some cases, only the month of symptom onset was available; in these cases, the 15th of each month was used as the basis for calculation.

### Imaging specifications

MRI was performed on 3 T scanner (Magnetom Prisma; Siemens Healthineers, Erlangen, Germany) using standard head–neck–spine 64 channel (3 T) coils. All participants included in this study received 3D fat‐saturated (fs) single slab turbo spin echo sequence with a slab selective, variable excitation pulse (Sampling Perfection with Application‐optimized Contrasts using different flip angle Evolution [SPACE] fs; repetition time: 1500 ms; echo time: 221 ms; flip angle: 115°; generalized autocalibrating partially parallel acquisitions [GRAPPA] factor = 2; 0.7x0.7x0.6 mm voxels; 88 contiguous sagittal slices, 0.63 mm).

In patients, the standard protocol furthermore contained sagittal 2D T1‐weighted (T1w) turbo spin echo (TSE) fs sequence of the lumbar spine after contrast (repetition time: 747 ms; echo time: 12 ms; flip angle: 150°; GRAPPA factor = 2; 0.68x0.68x2.2 mm voxels; 15 contiguous sagittal slices, 2.2 mm).

T2 SPACE fs was assessed independently by two radiologists (N.L., C.Z.) in all cases, blinded to the groups (cPDPH or controls), clinical data, and prior MRI results.

Since post‐contrast sequences were only conducted in the patient cohort, group blinding was not feasible. Still, the data were independently analyzed, blinded to patient information. In all cases of disagreement, a consensus was reached.

### Evaluation of the dinosaur tail sign

The presence of dinosaur tail signs was assessed as defined in the original study by Sakurai et al.^16^ with positive rating if more than half of the spinal level was covered with arched, continuous T2w hyperintensity. According to the original study, only imaging signs between Th11 and S3 were included in final analysis. Furthermore, for each case, the total number of levels showing those T2w hyperintensities and whether the sign was present over several consecutive spinal levels (consecutive) was recorded, whereby a subdivision was made: presence of the sign partly in consecutive levels and partly in distant levels (partially consecutive), or only in remote levels (non‐consecutive).

To increase precision, the subjective overall impression of the presence of the sign was evaluated as: (1) subtle, if the evaluator needed longer to decide on the presence of the sign; (2) moderate, if the presence of the sign was unequivocal and the decision was made without delay; and (3) prominent, if the evaluator was surprised by the strong expression of the sign.

### Evaluation of arachnoid blebs and epidural lumbar fluid

Assessment of arachnoid blebs and epidural lumbar fluid collections was made based on heavily T2w 3D high resolution imaging only to ensure blinded evaluation. For this, axial reformations were used to search for suspicious findings and then correlated with sagittal and coronal slices, if necessary. For blebs, the criterion for positive evaluation was defined as a round‐ovoid shaped T2w hyperintense structure at the dorsal aspect of the dura, protruding beyond the dural level. Another criterion was clear distinction from vascular structures.

### Evaluation of contrast enhanced lumbar MRI

Contrast enhanced T1w imaging of the lumbar spine in the patient cohort was first analyzed for any suspicious effacement. If present, this was further divided in dural enhancement (focal or multisegmental) and enhancement associated to nerval structures (focal, longitudinal mononerval, longitudinal multinerval and internerval). The criterion to call enhancement positive was a clear distinction between vascular structures or other clearly identifiable pathologies such as tumor lesions or facet joint inflammation.

### Statistical analysis

No statistical power calculation was conducted prior to the study. The sample size was based on the available data. The assumption of normal distribution was tested with the Shapiro–Wilk test. Descriptive analysis was performed using counts and percentages for categorical variables and mean (±SD) for normally distributed continuous data or median [interquartile ranges] for skewed data. Group analyses were assessed with independent samples *t*‐tests, Mann–Whitney *U*, Fisher's exact test, and chi‐squared test as appropriate. To account for potential confounding by demographic differences between groups, multivariable logistic regression analyses were performed for binary imaging outcomes, including group status, age, and sex as covariates. Effect estimates from these multivariable models are reported as adjusted odds ratios (aOR) to distinguish them from crude, unadjusted odds ratios (OR) derived from simple group comparisons. To provide unadjusted comparisons alongside the multivariable model, univariable logistic regression models were additionally performed for each covariate individually (group, age, sex). For the multivariable logistic regression model, assumptions were verified prior to interpretation. Multicollinearity was assessed using variance inflation factors (all VIF <1.4), and linearity in the log odds for age was assessed using the Box‐Tidwell approach (*p* = 0.845), with model fit confirmed by the Hosmer–Lemeshow test (*p* = 0.405). As all assumptions were met, no transformations or robust methods were required. Interrater agreement between the two independent readers was assessed using Cohen's kappa coefficient for presence of the dinosaur tail sign as well as contrast enhancement.

Sensitivity and specificity of the dinosaur tail sign for detection of cPDPH were calculated using a four‐fold table. Corresponding 95% confidence intervals (CI) were determined using the Wilson method. All hypothesis tests were two‐tailed and considered significant at an alpha level of 0.05. Benjamini–Hochberg false discovery rate (FDR) correction was applied to the family of primary group comparisons regarding imaging findings between patients and controls, comprising presence of fluid, blebs, presence of the dinosaur tail sign, number of affected levels, consecutive level involvement, and dinosaur tail sign grading. Raw (p) and adjusted *p*‐values (*p*‐adjusted) are reported separately.

Statistical analyses were performed using jamovi version 2.5 (The jamovi project, Sydney, Australia) and R version 4.4.1 (R Foundation for Statistical Computing, Vienna, Austria) in the RStudio 2025.09.2 + 418 environment (Posit PBC, Boston, MA, USA).

## RESULTS

Between September 2022 and December 2024, a total of 71 patients were scanned with our standard MRI protocol for PDPH. Of these, nine were excluded because of acute PDPH (symptom duration ≤14 days) and further eight patients due to prior spinal surgery in an external hospital. This resulted in 54 patients being included in the final analysis (Figure 1). Three of the included patients waived contrast enhanced MRI examination, resulting in missing data for the assessment of spinal contrast enhancement. In addition, brain MRI was unavailable for one patient. Apart from these, there were no missing data.

**FIGURE 1:**
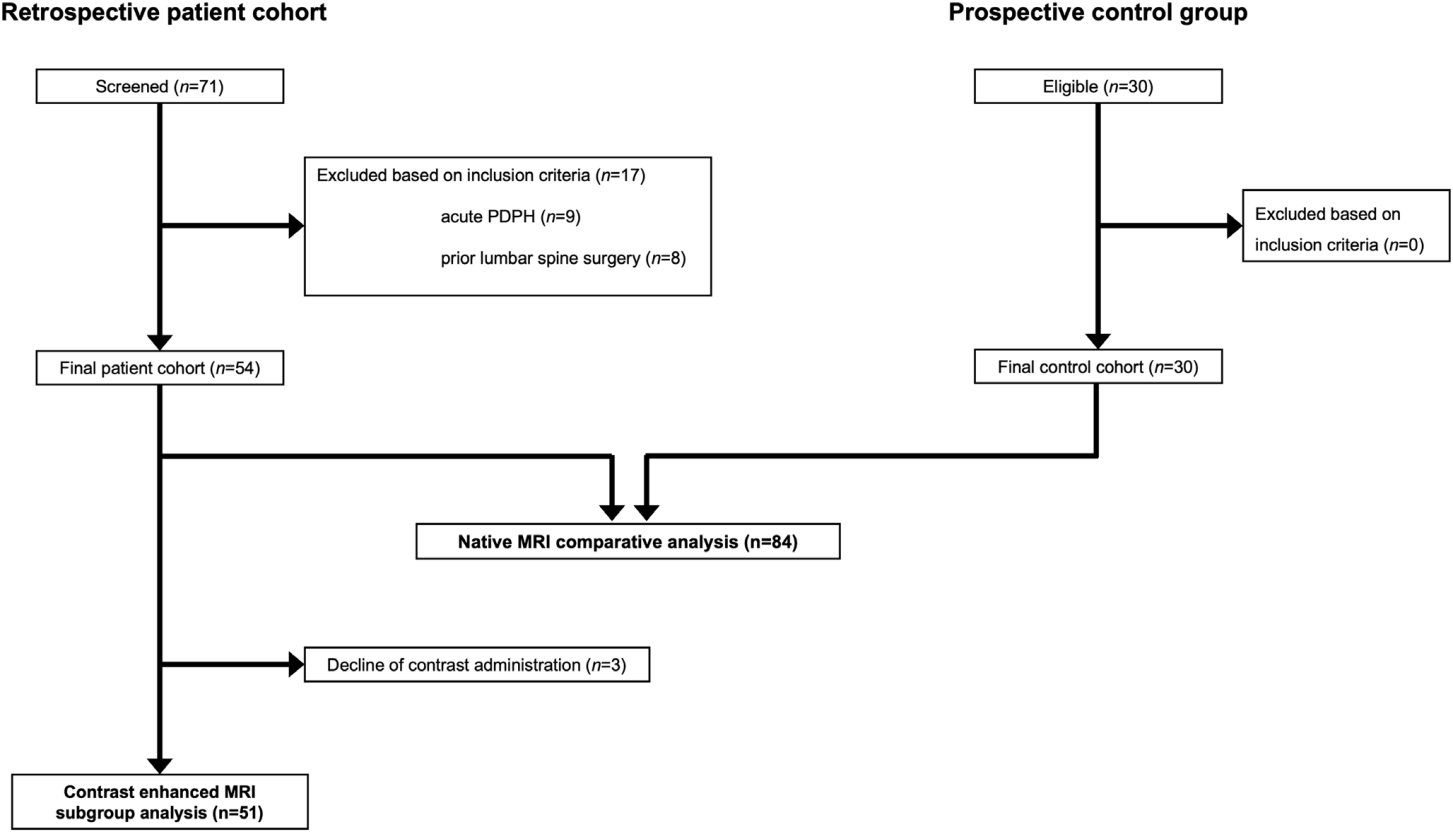
Flow chart of the study population. A total of 71 patients were investigated in our department with our dedicated PDPH protocol. After applying inclusion criteria, the final patient cohort resulted in 54 patients with chronic PDPH. A group of 30 healthy participants was prospectively recruited as controls and screened according to predefined exclusion criteria. The primary analysis compared native spinal MRI findings between patients and controls. A separate secondary analysis was performed in the subset of patients with available contrast‐enhanced imaging; no control comparison was available for this analysis. Three patients did not undergo contrast‐enhanced imaging. MRI, magnetic resonance imaging; PDPH, post–dural‐puncture headache.

### Demographic data

Of 54 patients with cPDPH, 44 (81%) were female, mean age was 38.2 years (±9,8, range 23–69 years), and mean BMI was 23.4 (±4.3, range 16.2–37.2).

Median symptom duration was 211.0 days (interquartile range [IQR] 110.0–407.0, range 28–2610). Median Bern Score (53/54) was 1.0 (IQR 1.0–3.0, range 0–9). 38/54 patients (70%) had received one or more EBPs before spinal imaging with a median of two EBPs (IQR 1.0–2.8, range 1–8).

The healthy control group consisted of 15 females and 15 males with a mean age of 29.5 years (±6.6, range 18–46) and mean BMI of 23.3 (±3.8, range 17.6–33.9).

### Imaging findings of the lumbar spine

#### Epidural fluid collections and arachnoid blebs

Epidural fluid collections in the lumbar spine were found in 4/54 patients (7%) vs. none in the control group (*p* = 0.292, *p*‐adjusted = 0.432). There were no discrepancies in the independent assessment of spinal fluid collections.

In total, blinded analysis of the lumbar spine MRI revealed 11 arachnoid blebs in 10 patients of the patient cohort (19%) with one patient presenting with two suspected blebs, but in none of the controls (*p* = 0.012). This association did not remain statistically significant after FDR correction (*p*‐adjusted = 0.060).

Consensus was needed in four MRI studies with substantial interrater agreement between both readers (*κ* = 0.78, *p* < 0.001, 95% CI 0.62–0.94). Overall, the bleb was rated as definite in 5/54 (9%) and as suspicious in 5/54 patients (9%), including the one patient with two suspected blebs on different levels. Of these, six patients had surgical exploration after MRI imaging (three of definite and three of suspicious subgroup). In five instances (three patients from definite subgroup, two from suspicious subgroup), surgery proved the presence of an arachnoid bleb in the suspected height, while the arachnoid bleb could not be identified intraoperatively in one patient. Additionally, the blinded analysis missed a bleb in three patients, which were confirmed at surgery. Demographic and imaging data of the patient cohort are summarized in Table 1.

**TABLE 1:** Demographic data and imaging features of the study cohort.

Variable	Available n	Patients (*n* = 54)	Controls (*n* = 30)	*p* (raw)	*p*‐adjusted
Age, years (mean [SD])	84	38.2 [9.8]	29.5 [6.6]	<0.001	
Sex, *n* (%)	84			0.002	
Female		44 (81%)	15 (50%)		
Male		10 (19%)	15 (50%)		
Body mass index, kg/m^2^ (mean [SD])	84	23.4 [4.3]	23.3 [3.8]	0.996	
Symptom duration, days (median [IQR])	54	211.0 [110.0, 407.0]			
Bern Score (median [IQR])	53	1.0 [1.0, 3.0]			
Bleb, *n* (%)	84			0.012	0.060
No		44 (81%)	30 (100%)		
Definite		5 (9%)	0 (0%)		
Suspected		4 (7%)	0 (0%)		
Double		1 (2%)	0 (0%)		
Enhancement, *n* (%)	51				
No		34 (67%)			
Ye		17 (33%)			

*Note*: The final cohort consisted of 54 patients with cPDPH and 30 healthy controls. Continuous variables are reported as mean ± SD or median [IQR], as appropriate. Categorical variables are reported as *n* (%). Available n refers to the number of non‐missing observations for each variable. Raw *p*‐values were calculated using *t*‐tests for normally distributed continuous variables, Mann–Whitney *U* tests for non‐normally distributed continuous variables and chi‐squared test or Fisher's exact tests for categorical variables as appropriate. Adjusted *p*‐values were calculated using the Benjamini–Hochberg correction across the family of six primary group comparisons.

Abbreviation: cPDPH, chronic post–dural‐puncture headache; IQR, interquartile range.

#### Analysis of the dinosaur tail sign

The dinosaur tail sign was found in 27/54 patients (50%) vs. 21/30 controls (70%). Therefore, the frequency of the dinosaur tail sign was higher in healthy controls but did not differ significantly (OR 2.31, 95% CI 0.83–6.84, *p* = 0.107, *p*‐adjusted = 0.240). After multivariable logistic regression analysis including group, age, and sex, group affiliation was still not independently associated with the presence of the dinosaur tail sign (aOR 1.53, 95% CI 0.45–5.52, *p* = 0.503). Age was independently associated with the presence of the dinosaur tail sign (aOR 0.89 per year increase, 95% CI 0.82–0.95, *p* < 0.001). Male sex was not significantly associated with the presence of the sign compared with female sex (aOR 0.32, 95% CI 0.09–1.03, *p* = 0.062).

Overall, consensus decision was needed in 20/84 MRI studies (23.8%), resulting in substantial agreement (*κ* = 0.61, *p* < 0.001, 95% CI 0.40–0.81).

A median of two levels was affected in both patient and control groups (IQR in patients 2.0–4.0, IQR in controls 1.0–3.0, range 1.0–6.0 each) without statistical difference (*p* = 0.289, *p*‐adjusted = 0.432). The respective distributions along the spinal levels in the two groups are visualized in Figure 2.

**FIGURE 2:**
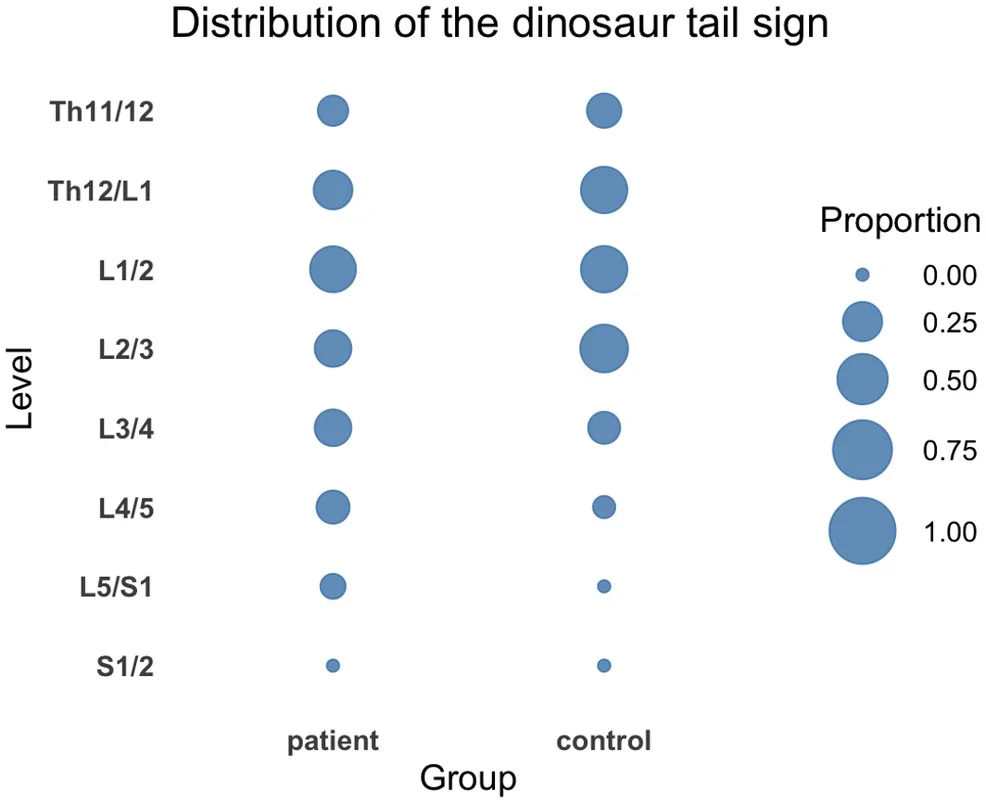
Balloon plot showing distribution of the dinosaur tail sign along the spine. Each tile represents the proportion of individuals in the respective group who showed a positive finding at the indicated vertebral level. Size of the circles encode the proportion of positive instances from small (0.0 = no positive findings) to big (1.0 = all individuals positive), with intermediate size indicating partial prevalence within the group. [Color figure can be viewed at wileyonlinelibrary.com]

In the subgroup rated positive for the dinosaur tail sign, the sign was mostly partially consecutive (12/27 (44%) patients vs. 8/21 (38%) controls). The consecutive or non‐consecutive morphology of the sign was not able to distinguish between the two groups (*p* = 0.778, *p*‐adjusted = 0.780). In both patient and control groups, the sign was most often rated “subtle” (15/27 [56%] patients; 15/21 [71%] controls). None of the controls had a “prominent” sign in contrast to three patients (11%), of whom two were also positive for epidural fluid collections in the lumbar spine (Figure 3). Notably, the third case without epidural fluid collection had the prominent appearance at only one spinal level.

**FIGURE 3:**
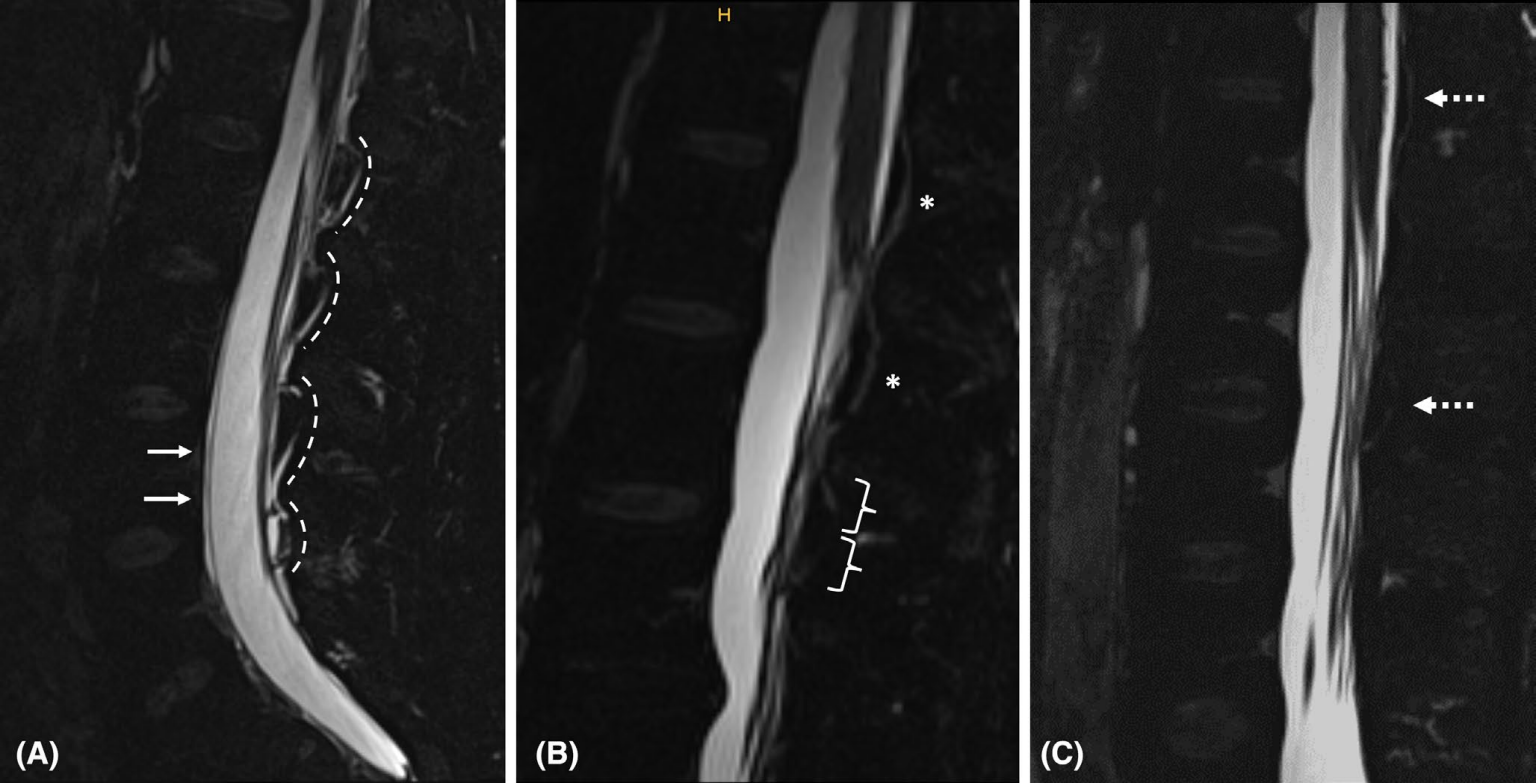
Evaluation of the dinosaur tail sign. (A) Lumbar spine MRI, positive for ventral and dorsal epidural fluid (arrows) in a 32‐year‐old female after unintended dural puncture during peridural anesthesia. T2‐weighted SPACE fat‐sat at 0.63 spatial resolution shows bright hyperintensities in the dorsal interspinous fat tissue on five consecutive levels (dotted arch), resembling the fins of a dinosaur which were described as the dinosaur tail sign. The global impression was graded as “prominent”. (B) Example of a female control, representing a “moderate” rated dinosaur tail sign at levels Th12/L1 and L1/2 (asterisks). One level below, slight arched hyperintensities in the dorsal epidural fat tissue are visible, however not covering more than 50% of the level or dorsal arch (bracket) and therefore to be interpreted as negative. (C) Lumbar spine MRI in a 31‐year‐old male patient with chronic post–dural‐puncture headache shows a “subtle” dinosaur tail sign at two spinal levels (dotted arrows). The level in between does not show the sign, this case is therefore interpreted as non‐consecutive appearance of the sign. L, lumbar; MRI, magnetic resonance imaging; Th, thoracic. [Color figure can be viewed at wileyonlinelibrary.com]

Consensus regarding the decision if an individual spinal level is interpreted as positive or negative was needed in 20 MRI studies (11 patients and nine controls), resulting in five decisions against the presence of dinosaur tail sign (three patients and two controls). If consensus resulted in positive reading of dinosaur tail sign, graduation of the sign was mostly subtle (15 studies) and moderate in three studies. In four controls, consensus for two levels was necessary. There were no discrepancies in the independent assessment among all patients with a prominent sign. Table 2 summarizes subanalysis of the dinosaur tail sign.

**TABLE 2:** Subanalysis of the different imaging features of the dinosaur tail sign in comparison between patients with cPDPH and healthy controls.

Variable	Patients (*n* = 27)	Controls (*n* = 21)	*p* (raw)	*p*‐adjusted
Levels (median [IQR])	2.0 [2.0, 4.0]	2.0 [1.0, 3.0]	0.289	0.432
Consecutive, *n* (%)			0.778	0.780
Yes	7 (26%)	8 (38%)		
No	8 (30%)	5 (24%)		
Partial	12 (44%)	8 (38%)		
Grading, *n* (%)			0.321	0.432
Subtle	15 (56%)	15 (71%)		
Moderate	9 (33%)	6 (29%)		
Prominent	3 (11%)	0 (0%)		
Consensus, *n* (%)			0.034	
No	19 (70%)	14 (67%)		
Single	8 (30%)	3 (14%)		
Double	0 (0%)	4 (19%)		

*Note*: Besides counts of positive levels, the sign was further evaluated regarding to its appearance: Presence of the sign only in consecutive levels (consecutive); partly in consecutive levels and partly in distant levels (partially consecutive), or only in remote levels (non‐consecutive). Furthermore, the subjective overall impression was noted (subtle, moderate, prominent). All data were analyzed blinded to patient information and independently. In case of disagreement, a consensus was reached.

Abbreviations: cPDPH, chronic post–dural‐puncture headache; IQR, interquartile range.

In our study, the sensitivity of the dinosaur tail sign for detecting cPDPH was 50.0% (95% CI 37.1%–62.9%), and the specificity was 30.0% (95% CI 16.7%–47.9%). The positive predictive value (PPV) was 56.3% (95% CI 42.3%–69.3%), and the negative predictive value (NPV) was 25.0% (95% CI 13.8%–41.1%). The corresponding values for frequencies are shown in the four‐fold Table 3.

**TABLE 3:** Four‐fold table for calculation of sensitivity, specificity, and PPV and NPV of the dinosaur tail sign in our cohort with cPDPH with healthy controls as the reference group.

Parameter	cPDPH	Controls	Total
Dinosaur tail sign present	27	21	48
Dinosaur tail sign absent	27	9	36
Total	54	30	

*Note*: Diagnostic performance. Sensitivity: 50.0% (95% CI 37.1%–62.9%). Specificity: 30.0% (95% CI 16.7%–47.9%). PPV: 56.3% (95% CI 42.3%–69.3%). NPV: 25.0% (95% CI 13.8%–41.1%). 95% confidence intervals were calculated using the Wilson method.

Abbreviations: CI, confidence interval; cPDPH, chronic post–dural‐puncture headache; PPV, positive predictive value; NPV, negative predictive value.

#### Results of contrast enhanced imaging

Suspicious contrast enhancement was found in 17/51 (33%). In detail, we found focal enhancement of the posterior dura as a potential correlate for a former lumbar puncture in 1/51 (2%) and any kind of nerval enhancement in 16/51 patients (31%): 2/17 patients (12%) showed focal enhancement at the cauda equina, 8/17 patients (47%) longitudinal effacement of one fascicle, 2/17 patients (12%) showed longitudinal enhancement of more than one fascicles, and 7/17 patients (41%) presented with focal enhancement between the nerve roots (Figure 4). In one patient, focal as well as longitudinal enhancement of one fascicle was found, and in another both, longitudinal effacement of one fascicle and focal enhancement between the nerve roots were present.

**FIGURE 4:**
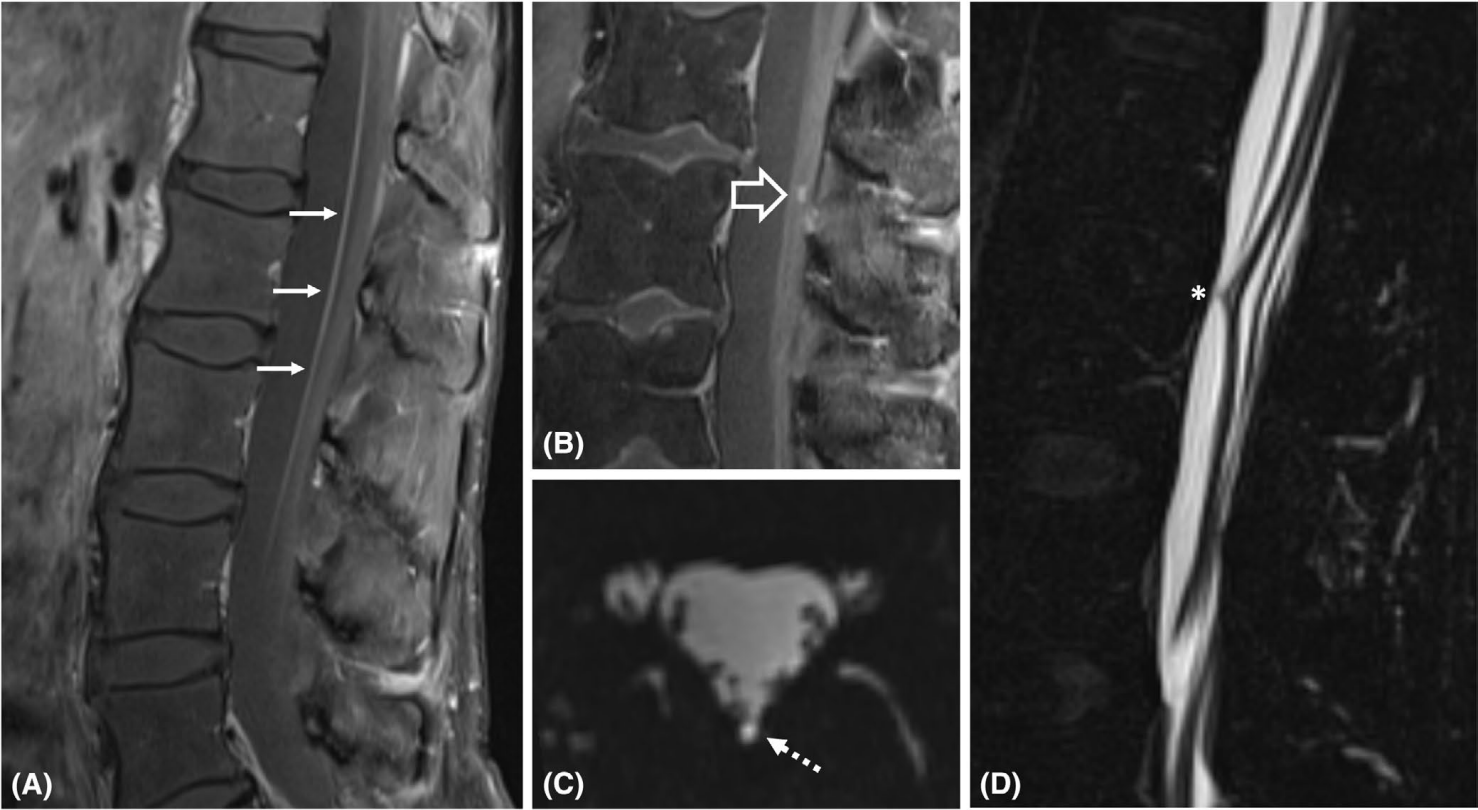
Imaging findings in the lumbar spine in cPDPH. (A) A 30‐year‐old female patient with history of unintended dural puncture during peridural anesthesia. Lumbar MRI after contrast administration shows longitudinal enhancement of the L4 fiber (arrows). (B) MRI in a 55‐year‐old male patient reveals an internerval focal contrast enhanced lesion (outlined arrow) at level L1/2. (C) 67‐year‐old female with post–dural‐puncture headache after lumbar puncture, who initially presented with subdural hematomas. At the dorsal site of the dura, there is a bright, round shaped T2w hyperintensity (dotted arrow), which represents a small herniation of an arachnoid layer through the dural puncture site, so called “arachnoid bleb” on Level L4/5. The finding was confirmed in a subsequent CT scan with intrathecal iodine contrast agent. (D) A 39‐year‐old female with cPDPH shows a ventral displacement of the cauda fiber (*), which seems to adhere to the ventral dura. cPDPH, chronic post–dural‐puncture headache; CT, computed tomography; L, lumbar; MRI, magnetic resonance imaging; T2w, T2 weighted.

Suspect contrast enhancement in the dorsal fat tissue (e.g., as a trajectory from a prior puncture) could not be identified in a single case. Consensus decision regarding the presence of suspicious contrast enhancement was necessary in 10 patients with six instances where the consensus led to positive evaluation of contrast enhancement and four patients where consensus led to negative interpretation of contrast enhancement, resulting in moderate agreement (*κ* = 0.57, *p* < 0.001, 95% CI 0.30–0.84).

## DISCUSSION

In this study, the dinosaur tail sign was found in both patients with cPDPH and, more often, in healthy controls, without significant difference, (*p* = 0.107) and was clearly not suitable as a diagnostic marker for cPDPH. This observation remained consistent after multivariable regression analysis. Other findings on spinal MRI, such as lumbar epidural fluid collections as an indicator of a dural tear and arachnoid blebs were exclusively found in cPDPH, but only occurred in a minority of patients. Furthermore, one‐third of all patients showed noticeable but mostly unspecific contrast enhancement in the lumbar spine.

Since the first description in 2017 in a single case report, the dinosaur tail sign was further evaluated in a comparative study between patients with SIH, with iatrogenic CSF loss after lumbar puncture (i.e., PDPH) and controls.^15^, ^16^ Here, the dinosaur tail sign was common in patients with SIH and acute PDPH; however, almost all of those cases were accompanied by spinal epidural fluid collections.^16^ This is consistent with the results of our study, in which all participants with epidural fluid were also positive for the dinosaur tail sign with a moderate or prominent overall image impression. It seems conceivable that epidural fluid collections, when distributed around the spinal canal due to an underlying dural tear, also result in fluid retention between the posterior ligament and the dorsal fatty tissue, which then becomes visible as T2w hyperintensities in the shape of arches. In contrast to acute PDPH, where epidural fluid is common,^16^ patients with chronic PDPH do not usually have epidural fluid accumulation, which may be result of resorption process over the course of time;^8^, ^14^ however, to date, the temporal evolution of epidural fluid collections in the course of PDPH remains incompletely understood. Still, the dinosaur tail sign was found quite frequently in patients (50%) and even more among the healthy controls (70%) of our study.

Since Sakurai et al. found the dinosaur tail sign in only two of 35 cases in their control group, the initial publication of this sign raised hopes that it could serve as a specific marker of CSF loss in PDPH, where the sign was present in 83% of cases. It was therefore interpreted as subtle extravasated epidural fluid collections in patients with PDPH.^16^ Our results, however, differ from those of Sakurai et al., and we were not able to distinguish between patients with cPDPH and controls applying the dinosaur tail sign, which may be due to the following reasons:

While applying the same criteria for the interpretation of the sign as described in the original study, we exclusively used high spatial resolution 3D heavily T2w SPACE fat‐saturated images at 0.63 mm spatial resolution (compared to 3‐ to 4‐mm fat‐saturated T2w images), which provides substantially higher spatial resolution. As a consequence, even subtle epidural hyperintensities and small venous structures that could be obscured in thicker slices may become conspicuous and contribute to the higher number of the dinosaur tail sign in controls in our study. Besides fluid retention, dilated epidural veins have also been discussed as underlying causes of this sign.^16^ We hypothesize that when the sign appears subtle or moderate, it could possibly be caused predominantly by dilated or prominent veins and not by free‐flowing epidural fluid. Therefore, the high proportion of the sign observed in the control group, predominantly presenting as subtle findings, may be attributable to subtle epidural venous structures that became detectable only due to the high spatial resolution of the MRI sequence used in our study; however, grading of the sign was also not able to distinguish between the two groups. To further evaluate this observation and to distinguish between epidural fluid and venous distension, a comparative study on the dinosaur tail sign exclusively in patients with SIH and CSF–venous fistula (that lack a dural tear) vs. healthy controls could be of great interest.

An additional important factor is that our control group was not matched for sex and age. Given that the pathophysiology of the dinosaur tail sign remains incompletely understood, the extent to which these demographic imbalances may have influenced our findings cannot be fully determined. Age‐ and sex‐related differences in connective tissue composition, venous anatomy, or hormonal influences could affect the appearance of the sign. While sex was not independently associated with the presence of the dinosaur tail sign, younger individuals showed higher odds of the sign, which may have contributed to the high proportion of positive findings in our relatively young control group; however, after adjustment for demographic differences, our results remained stable.

Furthermore, a relevant proportion of patients have undergone one or more EBP before MRI. Blood injected into the epidural space may induce local clot formation and scarring and, therefore, could possibly affect epidural fluid distribution as well as appearance of the dinosaur tail sign.^21^ However, to date, the extent to which prior EBP influences presentation of spinal imaging signs in cPDPH remains uncertain.

In general, patients with cPDPH may differ from other patients with spinal CSF leaks, as they appear to show fewer imaging findings currently considered typical markers of CSF loss, including spinal epidural fluid collections and cranial MRI signs summarized in the Bern Score.^8^, ^11^, ^14^, ^22^ Ultimately, the underlying pathomechanism in patients with cPDPH, sometimes causing severe symptoms of CSF hypotension, is unclear.^23^

One strength of our study is that we applied additional criteria such as number of levels, consecutive vs. non‐consecutive occurrence, and an additional subjective image impression beyond the mere presence of the sign. With this, we aimed to establish specific criteria such as cutoffs for the diagnosis of cPDPH that are also easy to implement in clinical practice; however, none of these criteria could differentiate between patients and controls.

In line with previous studies, the rate of epidural fluid collections in cPDPH was very low.^8^, ^14^ Besides fluid collections, a prior study from Schievink et al. found arachnoid blebs in 3/27 patients.^14^, ^23^ Our study complements those numbers by providing additional data on their incidence. In our cohort, we found suspected arachnoid blebs on imaging in 19%, of whom about 72% could be correlated intraoperatively. Notably, the blinded analysis of spinal MRI missed three surgical confirmed arachnoid blebs. One reason for this may be that group blinding led to a more restrictive interpretation and therefore only clear findings were evaluated as positive. In contrast, the decision for surgery on those three patients was made by an interdisciplinary board based on a review of clinical and imaging findings. This emphasizes the importance of combining clinical and MRI findings, and underscores the subtlety of arachnoid blebs, meaning that they can be overlooked even by experienced radiologists and at high spatial resolution imaging. This makes it even more important to pay close attention to these findings as they present a possible target for therapy.

As neither epidural fluid collections, nor arachnoid blebs were found in any of the healthy controls, they could represent specific detections for the presence of cPDPH; however, the statistical evidence from our data remains limited. In one patient, we furthermore observed a ventral displacement of a nerve fiber with obvious attachment to the ventral dura, suggestive of scarring adhesion following a lumbar puncture, which appears to be rare but specific.^24^, ^25^, ^26^

Our initial hypothesis was that lumbar puncture could lead to local scar tissue on the affected dura at the site of the former puncture, which may be visualizable using contrast‐enhanced imaging, potentially providing a specific target for treatment;^20^ however, our results show focal contrast enhancement of the dura in only one of all patients. Interestingly, a minor proportion (20%) of the patients did show suspicious contrast enhancement of cauda fibers or in 14% in between the cauda fibers, which might possibly be associated with granulation tissue or reactive changes following a dural puncture. Lately, these findings are nonspecific and cannot point to a specific pathological target (which could be addressed, for example, in exploratory surgery). Since we do not have a control group for contrast‐enhanced spinal MRIs, the results cannot be conclusively interpreted and should be further investigated in future studies; however, these are the first results of spinal imaging using contrast enhanced sequences in patients with cPDPH.

### Limitations

Our study has several limitations: First, the retrospective, monocentric design introduces inherent limitations, such as the inability to infer causality and the risk of selection bias regarding both patient inclusion and treatment approaches. Although we attempted to minimize selection bias by including all consecutive patients who fulfilled the inclusion criteria, the single‐center data collection likely resulted in a cohort representing particularly severe, complex, and treatment‐resistant cases of cPDPH, which may not be representative of the broader cPDPH population. Also, by comparing patients with cPDPH against healthy volunteers, the reported sensitivity, specificity, PPV, and NPV cannot be fully generalized to the clinical setting in cases where these imaging signs would need to discriminate between competing diagnoses.

Second, the study may have been insufficiently powered to detect clinically meaningful differences in rare findings such as arachnoid blebs and epidural fluid collections; accordingly, the non‐significant comparison of the dinosaur tail sign should be interpreted in the context of this uncertainty. Furthermore, as only a subset of suspected blebs underwent surgical confirmation and three surgically confirmed blebs were not identified on blinded MRI review, the true sensitivity of MRI remains indeterminate and the reported prevalence likely represents an underestimate.

Third, the control group and patients are not matched for age and sex, which could also influence the results and comparability. Nevertheless, the findings of our study remained after multivariable logistic regression.

Fourth, the effect on prior EBP, which have been performed in almost half of the patients before presentation at our clinic, on the interpretation of MRI signs is unknown.

To our knowledge, this is the largest cohort to date to investigate these specific topics.

## CONCLUSION

In this study, the dinosaur tail sign, originally described in acute PDPH, was unable to reliably distinguish patients with cPDPH from controls. Findings such as arachnoid blebs or extradural fluid were exclusively observed in cPDPH, but rare. As cPDPH is increasingly recognized and accompanied by tremendous symptom burden, further imaging signs and research is warranted to identify those patients and understand the underlying pathomechanism.

## AUTHOR CONTRIBUTIONS

**Charlotte Zander:** Conceptualization; methodology; data curation; validation; formal analysis; visualization; writing – original draft; writing – review and editing; investigation. **Horst Urbach:** Conceptualization; project administration; writing – review and editing; resources. **Katharina Wolf:** Resources; writing – review and editing. **Amir El Rahal:** Resources; writing – review and editing. **Florian Volz:** Resources; writing – review and editing. **Christian Fung:** Writing – review and editing. **Laura Krismer:** Writing – review and editing. **Hansjörg Mast:** Resources. **Jürgen Beck:** Project administration; writing – review and editing. **Niklas Lützen:** Conceptualization; methodology; investigation; supervision; writing – original draft; writing – review and editing; project administration.

## FUNDING INFORMATION

The authors have nothing to report.

## CONFLICT OF INTEREST STATEMENT

**Charlotte Zander, Katharina Wolf, Amir El Rahal, Florian Volz, Christian Fung, Laura Krismer, Hansjörg Mast, Jürgen Beck, Horst Urbach**, and **Niklas Lützen** have no competing financial and/or non‐financial interests in relation to the work described.

## CLINICAL TRIAL REGISTRATION NUMBERS

DRKS00034978; DRKS00035419.

## Supporting information


**Table S1:** Univariable and multivariable logistic regression for the presence of the dinosaur tail sign. OR = unadjusted odds ratio (univariable); aOR = adjusted odds ratio (multivariable model including group, age, and sex); CI = confidence interval. Reference categories: patient (Group), Female (Sex).
