# Supplementary material for: Re‐evaluation of the dinosaur tail sign: Analysis of spinal magnetic resonance imaging signs in patients with chronic post–dural‐puncture headache
Source: Headache. 2026 Sep 28;66(10):2432–42. doi: 10.1111/head.70210 (PMC13750328; doi:10.1111/head.70210)
Supplement: Supplementary file 1 [file HEAD-66-2432-s001.docx]

|  | **Multivariable analysis** | | | |  |  |  |  |
| --- | --- | --- | --- | --- | --- | --- | --- | --- |
| **Characteristic** | **N** | **OR (univariable)** | **95% CI** | **p-value** | | **aOR (multivariable)** | **95% CI** | **p-value** |
| Group | 84 |  |  |  | |  |  |  |
| patient |  | — | — |  | | — | — |  |
| control |  | 2.31 | 0.83, 6.84 | 0.107 | | 1.53 | 0.45, 5.52 | 0.503 |
| Age | 84 | 0.89 | 0.83, 0.94 | <0.001 | | 0.89 | 0.82, 0.95 | <0.001 |
| Sex | 84 |  |  |  | |  |  |  |
| Female |  | — | — |  | | — | — |  |
| Male |  | 0.59 | 0.23, 1.52 | 0.272 | | 0.32 | 0.09, 1.03 | 0.062 |

*Supplementary table 1: Univariable and multivariable logistic regression for the presence of the dinosaur tail sign. OR = unadjusted odds ratio (univariable); aOR = adjusted odds ratio (multivariable model including group, age, and sex); CI = confidence interval. Reference categories: patient (Group), Female (Sex).*
